# Resilient communities? A qualitative interview study on sustaining a community project for health promotion among socially disadvantaged women during the first two years of the COVID-19 pandemic

**DOI:** 10.1186/s12889-023-16593-9

**Published:** 2023-08-29

**Authors:** Sauter A., Linder S., Abu-Omar K., Sommer R., Herrmann-Johns A.

**Affiliations:** 1 Department of Epidemiology and Preventive Medicine, Medical Sociology, University of Regensburg, Universitätsstraße 31, 93053 Regensburg, Germany; 2 Department of Sport Science and Sport, Friedrich-Alexander-Universität Erlangen-Nuremberg, Gebbertstraße 123b, 91058 Erlangen, Germany; 3 School of Medicine and Public Health, HMRI Building, Level 4, West Wing, University of Newcastle, Callaghan, NSW 2308 Australia

**Keywords:** COVID-19, Physical activity, Community resilience, Health promotion, Participatory research, Low socioeconomic status, Qualitative research, Women’s health

## Abstract

**Objectives:**

With the emergence of SARS-CoV-2 a global pandemic impacted on health promotion, overlapping and hindering efforts to overcome the worldwide pandemic of lacking physical activity (PA). Nationwide lockdowns, the closure of public facilities and sports venues, made it significantly more difficult to sustain community-run PA projects. In our case study, we explore (a) under which circumstances a community-basedhealth promotion project can be maintained during crisis and (b) what resilience capacities are important for community project coordinators to deal with those aggravating circumstances.

**Methods:**

Our case study looks at a community-based project called BIG, an exercise promotion project for women in difficult life circumstances. The case study was conducted between July 2021 and January 2022 with six municipalities implementing the project. Following a triangulation approach, we used minutes from short exchanges (*n=*17) with community project coordinators, information brochures about current exercise classes (*n=*6) and semi-structured qualitative interviews with project coordinators (*n=*6). All data were analyzed using the framework approach.

**Results:**

All sites showed a high level of willingness to adapt to the pandemic situation and to maintain the project as best as possible. Findings highlight that coordinators whose work routine was characterized by intense relationship management with participants and trainers demonstrated higher levels of adaptive and absorptive capacities on an individual level than coordinators of those communities with less social cohesion. At a community level, important resources for strengthening adaptive and absorptive resilience capacities were job security of the coordinators, sufficient financial resources to adapt classes to changing circumstances, and a supportive organizational climate in the coordinators’ working environment to allow them to react flexibly according to current pandemic regulations.

**Conclusion:**

Despite high resilience capacities especially on an individual level, the low-threshold nature of the project could not be maintained at a pre-pandemic level. For many women, participation in the project was no longer possible at times. Awareness should be raised in communities that PA promotion programs are crucial to strengthening physical and mental health, even in times of crisis. It seems necessary to find permanent funding options for such programs, to integrate them permanently into municipal structures.

## Introduction

Regular physical activity (PA) is associated with numerous health benefits. A physically active lifestyle can be protective against the development of cardiovascular disease, diabetes, overweight and obesity, osteoporosis, and certain types of cancer, in particular colon and breast cancer [1]. Some of those non-communicable and chronic diseases are also associated with a severe course of COVID-19 [2–4]. The World Health Organization (WHO) recommends a minimum of 150 minutes of moderate-intensity endurance-based exercise per week or 75 minutes of higher-intensity endurance-based exercise per week for adults aged 18-65 years [5]. However, PA also has a positive effect on the immune system [6, 7]. Regular light to moderate PA can help improve immune function and generally reduce the risk, duration or severity of viral infections [8]. Several studies indicate the positive effect of regular PA on psychological well-being and mental health in times of social isolation, which is especially important for those groups living alone or in small households such as single parents [9, 10].

Promoting PA is an important public health goal across all population groups, but in particular for those most vulnerable such as those with lower socioeconomic status (SES), especially women with lower SES [11, 12]. Data from Germany, for example, show that women with lower SES are more physically inactive than women with higher SES and women with an immigrant background are less active than women without an immigrant background [13, 14]. Reasons for this can be family duties, lack of social support, low financial resources or a lack of culturally sensitive PA offerings for ethnic minorities [15–17].

However, with the outbreak of the COVID-19 pandemic, PA promotion programs have been restricted, although they are relevant for physical and mental health [18]. Especially during the first 18 months of the pandemic when no vaccination against a SARS-CoV-2 (Severe Acute Respiratory Syndrome Coronavirus 2) infection was available, public life restrictions, such as social distancing and closing public sport facilities, impeded group exercise classes [19]. In times of lockdowns, homeschooling, home-office and lack of childcare, made everyday life, work and family management even more exhaustive and stressful for many women, which also made exercising individually at home or outdoors difficult or not possible at all [20, 21].

### Health promotion in a moral dilemma

COVID-19 and physical inactivity are two overlapping pandemics which seemed to pose a moral dilemma for PA related health promotion. On the one hand, municipalities must follow federal regulations to prevent the virus from spreading and to protect vulnerable populations. On the other hand, this requires that exercise programs are cancelled, although they are beneficial for people’s physical and mental well-being and can also strengthen their immune system. Thus, due to the COVID-19 pandemic and the sudden lockdowns many communities faced an unexpected crisis given that existing PA and social activity offerings had to be stopped immediately. Also, uncertainties about the course of the pandemic, the sudden changes in work routines due to home office and digitalization or worries about job security were some of the barriers that administrative staff responsible for many PA projects in communities faced since the beginning of the pandemic [22].

### Community resilience as key approach during the crisis

From previous global and national crises we know that community resilience is an important resource for overcoming a disaster (e. g. floods, earthquakes) [23]. Community resilience can therefore become a key aspect for coping with the challenges occurring through the COVID-19-pandemic. Resilience is a multi-dimensional concept, describing the ability of individuals, communities or systems to cope with changes or crises, such as war, terrorism or environmental disasters [24]. Different definitions of resilience have been discussed, however, all definitions support the idea that resilience is linked to processes and capacities that promote health and health related behaviors for individuals and communities in times of crisis [25]. The WHO defines resilience as *“the ability to prepare for, manage (absorb, adapt and transform) and learn from shocks”* [26]. The WHO defines four types of resilience capacity which help individuals or communities to withstand and recover from unfavorable circumstances and to gain a sense of “being in control”: (1) adaptive: ability to adjust to shocks; (2) absorptive: ability to absorb and cope with shocks, manage and recover from unfavorable circumstances; (3) anticipatory: ability to predict and reduce disturbances; (4) transformative: ability to transform (system) structures [25, 27, 28]. These four capacities are a key aspect for effective public health policies and programs in order to promote health and health behavior in a society and to create conditions that reduce health inequities [29]. Only by constantly adapting programs to changing (societal and environmental) conditions and needs, the reach and effectiveness of a program can be guaranteed in the long term.

Yet, little is known about how communities have dealt with the sudden COVID-19 pandemic in in the first two years regarding the maintenance of PA promotion programs [30]. Previous research has focused mainly on the impact of the pandemic on scientific project work at research institutions [31, 32], the impact of the pandemic on the work routine of community health centers [33] or the role of community nurses and healthcare workers and their mental health [34, 35]. However, there is little research on how administrative community actors dealt with the sudden onset of the pandemic and how the crisis affected their daily work of sustaining a health promotion project. Thus, further evidence is needed to better support communities with maintaining project work during crisis which is important for the health of vulnerable groups. This case-study aims to address this research gap and to generate knowledge on communities’ resilience to better understand coping strategies to overcome crises and continue PA programs. These findings may help communities to better overcome future crisis and emergency situations.

To better understand strategies to sustain communal PA promotion efforts during the COVID19-pandemic this study explored:

- The impact of the COVID-19 pandemic on community project work to maintain PA offerings for women in a low-threshold and target-group sensitive manner.
- What strategies strengthened different types of resilience capacities of the project coordinators to maintain a project despite the crisis.

## Methods

### BIG as case study

This study explored the effects of the COVID-19 pandemic on the maintenance of a German community-based research project called BIG (Bewegung als Investition in Gesundheit – movement as investment in health) [36]. The BIG-project was developed by the Department of Sport Science and Sport of Friedrich-Alexander-Universität Erlangen-Nuremberg in Germany in 2005 [36, 37]. The aim of the BIG-project is to promote PA among women in difficult life situations (e.g., having a low household income, a migration background, being unemployed, relying on welfare aid, or being single mothers), by using a participatory approach. This means that women actively participate in the planning and implementation of low-threshold exercise classes (e. g., free of charge, availability of childcare, close to the places where women live). This also involves that the municipality is appointing a BIG coordinator who is responsible for project management and maintaining contact with the women.

Since 2005, BIG has been transferred to several sites in Germany. Out of those, four sites are currently starting to implement the project, in seven communities the project has been running successfully for several years. All communities are using the same approach which allows to compare the processes and effects of the pandemic in all sites.

Before the pandemic, across all sites, approximately 800 women regularly took part in about 60 different exercise classes [38]. BIG offerings at active sites included for example regular exercise classes, beginner bicycling classes, women-only swimming classes but also other events such as women breakfasts, cooking classes and a train-the trainer program [38].

The present study was conducted as part of a federally funded follow-up of the BIG-project, called NU-BIG which aims to assess the long-term effects of the BIG-project across all project sites. This project is described in detail elsewhere [39].

### Communities involved

Five communities in Bavaria and one community in Berlin were included in this case study to describe the effects of the COVID-19 pandemic on maintaining BIG at the different sites. These six sites were selected because they had been running successfully for several years prior to the COVID-19 pandemic, allowing a comparison of project work before and during the pandemic. At each site, the BIG coordinators involved in this study have been managing BIG for several years. Prior to the COVID-19 pandemic, all sites had an established range of needs-based exercise classes, and four of the six communities also offered other activities as part of BIG (e. g., women's breakfasts). More detailed information on the municipalities can be found in Table 1. The main target groups of these six municipalities were women with a migration background, women with a low income and women with mental health problems.

**Table 1:** Overview data-set included in the case study

	Project start	Employment of the project coordination	Interviewees per interview	Interviewees professional years in BIG	Interview procedure	Short exchanges (telephone calls, E-Mails)	Information brochures
**Community A**	2005	Municipality	*n=*1	3 years	Telephone *(11/2021)*	*n=*4 *(03/2021-04/2022)*	*n=*2
**Community B**	2008	Municipality	*n=*2	3 years	Video-call *(12/2021)*	*n=*2 *(10/2021-02/2022)*	*n=*2
**Community C**	2009	Local sport club	*n=*1	5 years	Telephone *(01/2022)*	*n=*2 *(04/2021-02/2022)*	none
**Community D**	2011	Municipality	*n=*1	12 years	Telephone *(07/2021)*	*n=*3 *(09/2020-10/2021)*	*n=*2
**Community E**	2013	Municipality	*n=*1	10 years	Video-call *(08/2021)*	*n=*3 *(06/2020-03/2022)*	none
**Community F**	2017	Local non-profit organization	*n=*1	5 years	Telephone *(08/2021)*	*n=*3 *(07/2020-02/2022)*	none

**Fig. 1:**
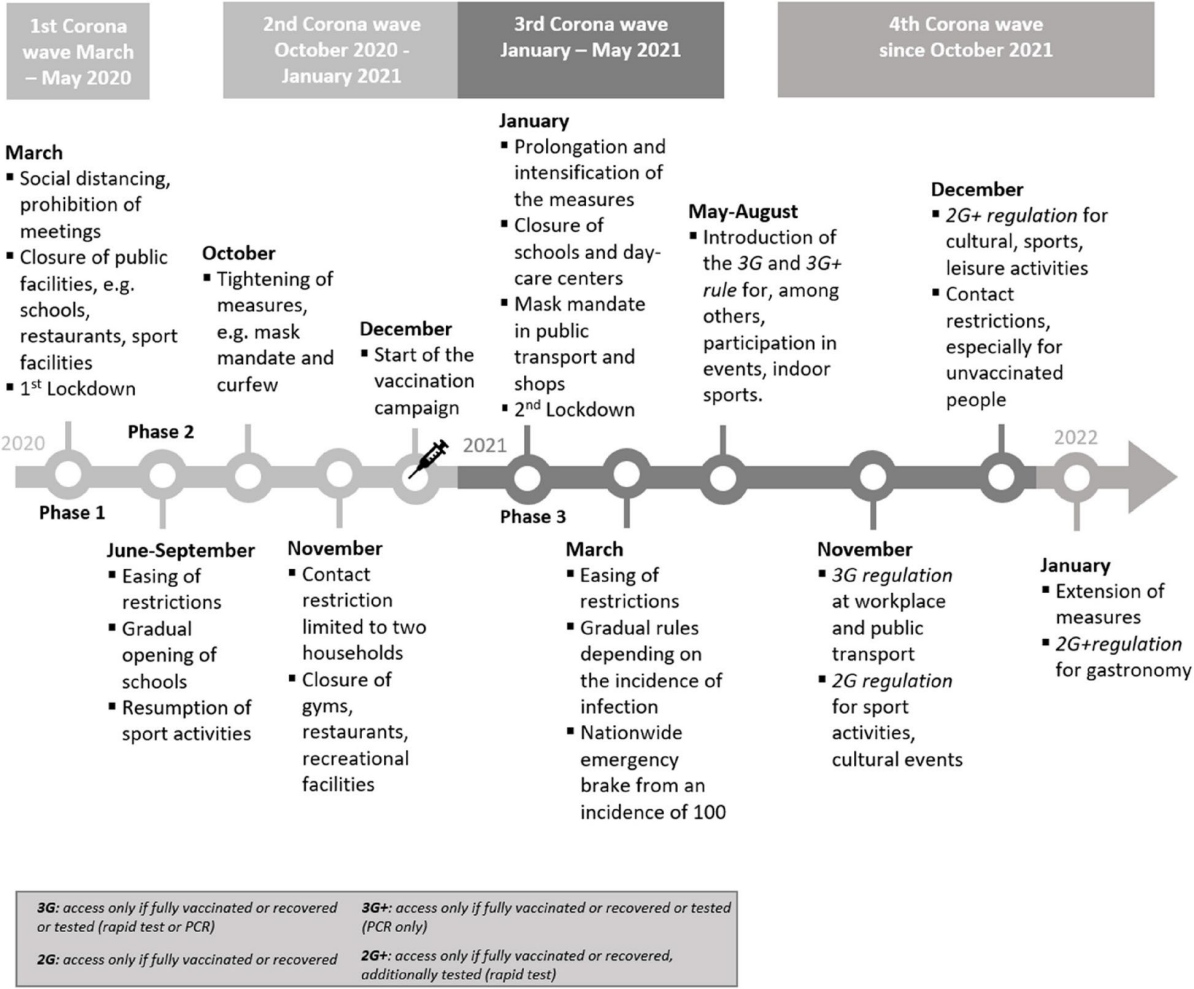
Overview COVID-19 restrictions Germany

The narratives in the interviews and the minutes from telephone calls cover the time period from March 2020, when the first lockdown occurred during the COVID-19 pandemic, to January 2022. Figure 1 shows an overview of the individual pandemic waves, lockdowns and federal measures to contain the pandemic during this timeframe in Germany. The measures shown in Fig. 1 focus on the regulation of sports facilities and the provision of group sports activities.

### Ethical considerations

The Ethics Committee of the Friedrich-Alexander-University Erlangen-Nuremberg granted ethical approval for this follow-up study (approval number: 247_20 B). All interviewees gave informed written consent for the interview, the audio recording and scientific use of their accounts.

### Data collection and analysis

We followed the recommendations of Mays and Pope for triangulating qualitative data to ensure rigor and quality management of data collection and analysis [40, 41]. Three data sources were used for the case study: Semi-structured qualitative interviews with project coordinators, minutes of short exchanges with administrative staff from the six project sites and information brochures from various sites, if available.

### Semi-structured interviews

Qualitative semi-structured interviews were held between July 2021 and January 2022 with project coordinators managing the BIG project in the various communities (*n=*6) via telephone or a video-based communication tool. Interviews lasted between 46 and 73 minutes (mean: 51 minutes). Questions of the used topic guide were based on Glasgow et al.`s RE-AIM framework, which provides a comprehensive framework for evaluating the impact of public health interventions [42]. This theoretical framework is used for the long-term evaluation of the BIG-project as it provides a systematic and comprehensive approach for evaluating the impact of a public health intervention [39]. The framework consists of the following five dimensions: reach, efficacy, adoption, implementation and maintenance. Table 2 gives an overview over the RE-AIM dimensions and the corresponding questions for each dimension. Interviews were audio recorded and transcribed verbatim.

**Table 2:** Main questions topic guide

**Domain RE-AIM**	**Definition**	**Question topic guide**
Reach	Can the target group be reached by the program?	• Which women took part in the BIG offers before the COVID-19 pandemic? • Which women have stopped coming to the BIG-activities since the COVID-19 pandemic? • What were the reasons why some women no longer participated in the offers? • To what extent could the activities still be offered at a low threshold since the COVID-19 pandemic?
Efficacy	What effect does the participation in the program have on the target group?	• What were the reasons why women have returned to the program after the Corona break?
Adoption & Implementation	Which determinants are responsible for a successful implementation of a program?	• What impact did the COVID-19 pandemic have on sustaining the BIG project? • What changes had to be made to the project? • What impact has the work as a BIG-coordinator on your well-being during the COVID-19 pandemic?
Maintenance	What factors determine the sustainable continuation of a program and the long-lasting commitment of the target group to a program?	• What resources are needed to continue to run BIG?

The transcripts were analyzed using framework analysis [43]. This approach is appropriate for mapping and interpreting qualitative data and helped the research team to develop an in-depth understanding of the coordinator’s experiences during the COVID-19-pandemic and the effect it had on their daily work related to maintaining the BIG-project. The following steps were undertaken for analysis: First, deductive coding was performed by using the coding software ATLAS.ti Version 8 in order to identify, sort and store relevant text passages. Each interview served as a unit of analysis and was coded line by line. The applied codes were related to the dimensions of the RE-AIM model as shown in Table 2 (e. g., the code “Closure of sports facilities as a security measure” was assigned to the maintenance dimension). In a next step, sets of codes were discussed with the research team to find commonalities, form an analytical framework and thus group them around larger themes, i. e., the various resilience capacities. On this basis, the analytical framework was formed. The analytical framework describes how the dimensions of RE-AIM were affected by different aspects of the COVID-19 pandemic. The framework was then applied to all transcripts. Afterwards themes (i. e., threads of meaning) were generated across categories by interpreting and explaining the categories and the associations between them. The themes referred to what resilience capacities were helpful in dealing with changes, e. g., in terms of continuing to run the exercise classes or maintaining contact with participating women. All developed themes and the conclusions drawn by them were reviewed by members of the research team to check whether themes were coherent and captured the most relevant features of the data.

### Short exchange

Since the beginning of the COVID-19 pandemic, the scientific staff of the University of Erlangen-Nuremberg has held regular telephone calls and e-mail catch-ups with the project coordinators at the individual sites to get a brief overview of the situation on site and to stay informed about the running of the project during the pandemic. These short exchanges provided a suitably complement to the qualitative interviews, as they confirmed information from the interviews and also provided further details about the situation at each site after the interview period. For this case study, a total of 17 documents of short exchanges from April 2020 to March 2022 were included. They were analyzed as additional data material with the established framework. Attention was paid to whether new information emerged from the minutes that could be used to answer the research questions and whether information was consistent or contradictory to the results of analyzing the interviews.

### Information brochures

Information material from sites that provided information about their current BIG program was included in the analysis as an additional data source. A total of six brochures from the two years of the pandemic were included. These provided information on the number of classes offered per semester, the type of classes (in-person, online, outdoor) and any restrictions due to the COVID-19 pandemic.

## Findings

The following section describes the influence of the COVID-19 pandemic on the maintenance of the BIG project at all six sites and the mechanisms, that have contributed to strengthen resilience at various sites. Against the background of WHOs understanding of resilience capacity [25, 29], we identified six themes that impacted the coordinators’ resilience to pursue positive changes within the communities. Adaptive resilience capacities: (1) guaranteeing job security for the coordinator, (2) enabling flexibility in working methods and processes, (3) maintaining contact with the target group. Absorptive and anticipatory resilience capacities: (4) maintenance of established local BIG networks, (5) availability of financial resources. Transformative resilience capacity: (6) changes of project structures without a permanent transition.

### Factors influencing the adaptive resilience capacities of BIG coordinators

During the first months of the pandemic in 2020, the adaptive resilience capacity of BIG coordinators was especially important to overcome the shock of the sudden pandemic, where all ongoing BIG classes and other social events such as the women breakfasts or meetings with project partners had to be stopped. Adaptive capacity helped the coordinators to retain the project’s basic work flow and functions in a best possible way despite the adverse circumstances.

#### Guaranteed job security for the coordinator

An important factor to strengthen coordinators’ self-efficacy and stress resilience during this period seemed to be whether the coordinators had job security, i. e., their employer was still in favor of the BIG project despite the pandemic situation and the possible breaks in the program continuation due to political regulations. Coordinators reported different situations in their communities. Some coordinators were worried whether their superiors (e. g. head of the respective administrative office) would lose interest in the project if the coordinators could not maintain contact with the target group during the pandemic and could no longer prove that the classes were well attended. Without the superior's support, the funding for the project and thus also for their job might fall away. Especially in municipality D and F, there was already a concern before the start of the pandemic that their positions as BIG coordinator could be cut (e. g., because the municipal budget could provide less money for social and health projects). These fears intensified with the emergence of COVID-19 and the resulting additional costs for the respective municipality. This seemed to lower their resilience capacity respectively their motivation to push the project even further. In communities A, B, C and E the respective coordinators had some more degree of job security, as the coordinators also had other responsibilities within their employment with the municipality. This increased their stress level during the pandemic, as they had more workload not only within BIG. However, it gave them a sense of security not to lose their employment during the pandemic. This sense of security strengthened their adaptive resilience capacity and had a positive impact on their work as coordinators. They reported being more relaxed about daily uncertainties because they did not feel they had to perform a certain way to please their superiors.

“I have all options. It's not like I have to make sure that I make a profit and survive, but this is a municipal project, I do my best, we see what we can implement [during the pandemic]. That's why it's not so bad”. (Interview community A)

#### Enabling flexibility in strategies and processes for course adaption

As the pandemic progressed in 2020 it was possible to stepwise reopen some classes outdoor or online. In the interviews and additional short exchanges, the project coordinators reported continuing great interest and high demand by the women who were wondering when the BIG classes would be resumed after the first lockdown. An important factor to strengthen the coordinators adaptive resilience capacity during this time was a high degree of self-organization and flexibility to improvise and to design daily work processes in accordance with the new demands of the pandemic situation.

“The women told us they won’t go on summer vacation [in 2020] as planned, and they want a [BIG] summer program so badly. That’s when we started improvising. I had to do everything anew: The graphic designer, the advertising, the room rentals. It’s insane. But we had a summer program for July, August, everything open air”. (Interview community A)

Having the opportunity to do field work to find suitable locations for outdoor classes, to prepare exercise rooms for online streaming or to promote the new classes after the first lockdowns, was reported to be necessary to adapt and thus maintain the project. Also, regular visits to new outdoor classes were considered important to strengthen the interpersonal connection with the participating women and to check whether classes were offered in a needs-based way.

However, this flexibility was not possible at all sites due to existing bureaucratic rules in some community administrations. For example, in community D the regular short-exchanges with the coordinator emphasizes difficulties regarding the use of video conference tools, as the community administration only provided limited access to licenses for an appropriate video conference software to stream online classes. Also, working in the evening was not allowed. Thus, it was not possible for the coordinator to offer online evening classes which made it difficult to transform the project in an appropriate way. Only community F did not have to make any adjustments, as the existing BIG course was mostly held outdoors anyway and they could continue with their course as usual after the first lockdown.

#### Maintaining contact with the target group helps retain motivation

The repeated reorientation and adaptation of classes seemed to require an abundance of time and energy from the coordinators. The uncertainties caused by the changing political requirements made their work feels like “*a walk on eggshells*” (Interview community E). Especially as political requirements tended to change often and on a short-notice. The coordinators reported that in some cases they had to pay attention not only to the federal COVID-19 regulations, but also to state-specific regulations and specific regulations of local sports associations. Furthermore, when children were present, different regulations applied than for courses for adults only, which made it even harder to adapt classes properly.

“We had to completely change the participant lists [for the BIG classes]. Normally, there is only first and last name. This had to be extended to enter the vaccination status. Then we had this contact tracking for a while, where the lists had to be kept for two weeks. Of course, this is all very time-consuming. In the sports club building, we have I don't know how many different access rules. Some applies to children, some applies to adults, some applies to employees, some applies to exercise instructors. So, it's madness”. (Interview community C)

An important factor to cope with the additional work load and permanent stress and to strengthen the adaptive resilience capacity was the great interest and high demand by women who were constantly inquiring when the BIG classes would be resumed after the various lockdowns in 2020 and 2021. Coordinators described this high demand as very encouraging for their work. Keeping in touch with the women during lockdowns, mainly through messenger services and phone calls, or by visiting them in the outdoor classes, was rated as important strategy to maintain the established trustful relationship with women and to be aware of their needs and demands. Interviewees reported that the rapid re-implementation of classes was highly appreciated by the women, which gave the coordinators energy and motivation to get through the months to come involving high workload and constantly changing restrictions.

“There is a very high appreciation from the women. Many of them write or call me and say they are so happy and that's so nice. And those are the moments when you know you did the right thing. There's someone who appreciates it and then it makes even more sense to work flat out”. (Interview community C)

### Factors influencing absorptive and anticipatory resilience capacities of BIG coordinators

As the pandemic progressed in late 2020 and 2021 the absorptive capacity of the BIG coordinators seemed to be very important to effectively cope with the ongoing COVID-19 pandemic and to adapt the BIG project in the best possible way. Using available skills and resources were important to manage and recover from the adverse conditions caused by the federal and states regulations to re-open exercise classes. Some of those resources seem further important to also strengthen the anticipatory capacity, the proactive action to reduce vulnerability to further unfavorable events.

#### Maintenance of established local BIG networks for re-opening and adapting BIG classes

Maintaining contacts with local partners who support the project, as well as with the trainers, was reported to be an important strategy in order to keep all key players involved and engaged for times when classes had to be re-opened and adapted quickly. However, this seemed to be rather difficult at some sites. Partners’ priorities seemed to not always support a PA project in times of the COVID-19 pandemic. Another difficulty was the identification and bonding with new contact persons. Also, some stakeholders seemed no longer as motivated and driven to achieve the best possible outcome for the project, due to increased workload, many throwbacks in the planning and implementation of classes in the past months and overall psychological impacts caused by the pandemic.

“There is also a sense of planning apathy among district centers [which offer BIG classes]. People don't really want to keep planning anymore, and there's a kind of corona blues. You don't have to do everything that you could do. A certain lethargy I would say”. (Interview community B)

Therefore, two communities which held regular cooperative planning meetings before the pandemic, tried to re-introduce them in a face-to-face format as soon as possible in 2021 to strengthen ties and to raise awareness of the project among stakeholders and trainers.

“[…] that the coordination teams meet in person from time to time. In other words, to stay on top of it. They never have time, and a video conference can be canceled more quickly than a face-to-face meeting. I think it's important that we manage to meet everyone in person at least once a year”. (Interview community B)

“[In 2020] we had to cancel one [planning group meeting]. The number of participants was extremely reduced. That would not have made any sense. In June [2021] we held the first planning meeting again. And that was a huge success. There were over 80 women there. We did it open air. We built a beer garden together with the [local] mosque. With the help of a lot of people, and we cooked for the women ourselves. It was really great”. (Interview community A)

Another important strategy was to maintain contact with the courses’ trainers. Many coordinators highlighted the importance of keeping the trainers informed about the current status and ensure them that they would be rehired as soon as classes could be held again. Otherwise, there would be a risk that trainers would look for other jobs to make a living or loose general interest in being a trainer.

“Suddenly people [trainers] simply do something else, a new job, or they say, ‘I actually like doing something with my granddaughter on the weekend. I don't need to give a course on the weekend anymore’. And that's why it was important that we were able to maintain most things [and stay in contact]. Also, because we have a responsibility towards the trainers, who rely on us and partly live from their teaching income”. (Interview community A)

Also, at some sites, trainers seemed to be an important vehicle to stay in contact with the women and to explain the current restrictions to them in their first language. This seemed to be an important resource for the coordinators for not losing participating women but bonding them to the project in times of lockdowns and online only classes.

“I was in constant contact with our trainer for the women's only swimming classes. She kept asking [what’s the status]. I updated her and she organized herself very well via WhatsApp groups with her course participants. She practically always kept the women up to date”. (Interview community C)

#### Availability of financial resources to adjust exercise classes

Implementing online classes caused significant financial strain on some sites, since licenses for video conferencing tools had to be purchased, as well as the technical equipment for broadcasting the classes (e. g., laptops, cameras, microphones). Although all BIG sites had a limited budget for extra spending, they felt there was no alternative to investing in the new equipment in order to be able to exist next to other sport offerings in their community and not lose participants to other online offerings. Also, having the possibility to offer online or hybrid classes was also an important facilitating factor for the community’s anticipatory capacities in order to proactively reduce further disturbances and to minimize vulnerability caused by new regulations.

“First of all, you need licenses for everything so the trainer has access [to Zoom®]. In some cases, the sports club didn’t even have this kind of [technical] equipment. They had to buy it first. But you grow with the challenge (…) I mean the sport club actually had to consider ‘Can we survive this?’ And then that’s an option you have to take [buying licenses, offering online classes]”. (Interview community C)

### Factors influencing the transformative resilience capacity

#### Changes of project structures to better fit to the new conditions

For most communities it was possible to react flexibly and adapt the BIG project in a way that was more suited to new pandemic-related requirements and restrictions (e. g., online, outdoor or hybrid classes). Therefore, the transformative capacity seemed important to transform the structures and means of operating of the BIG project to better address the pandemic related political changes and requirements. But it seemed difficult to permanently transform the design of the BIG project and at the same time maintain its health effects. Due to all changes made to fulfill legal requirements, most coordinators reported that the project lost its needs-based and low threshold character, which stopped some women to continue participating. Online offerings could not be taken up by some women, for example, due to a lack of technical equipment, childcare or insufficient private space for exercising at home.

“During lockdown there was already the changeover to online. But it was not accepted by the group. Maybe because you don't feel so comfortable at home. I mean, we had a lot of people who said, ‘I don't want to be running around in front of my family in the living room,’ or ‘at this time my husband needs the computer, because he has home office’. It was a possibility, but it was a 50-50 thing”. (Interview community C)

Outdoor classes seemed also uncomfortable for some because of the exposure to the public and lack of changing rooms.

"We didn't have as many participants as usual, of course. We noticed that not everybody likes outdoor. It was the heat, the rain, the mosquitoes, the noise. Then it was somehow because we were not allowed to use the public toilets. It wasn't as easy as we thought it would be”.* (Interview community A)*

In times where federal regulations prescribed permanent ventilation, which was mostly met by open windows and doors, classrooms were no longer protected from (male) views, which seemed to be a major barrier for class participation, especially for women of Muslim faith. Mandatory testing also seemed to be a major barrier to participation for many, especially for low-income women. Rapid tests were rated as expensive and at times hardly available in Germany.

"The need to do a test is also a financial issue. I mean these rapid tests cost four or five euros. So even if you were to say, ‘Okay, you try to make it low-threshold and do this self-test at the location’, even then it’s a financial issue. A test costs twice as much as the course hour. For our women that just plays a huge role”.* (Interview community B)*

The temporary requirement to be vaccinated also seemed to be a major barrier for some. In community E the coordinator reported in short exchanges, many women were not vaccinated, so it was not possible for the coordinator to offer classes during the autumn 2021 and winter season 2021/22.

#### Timely limited transformation – back to status quo ante

Therefore, at all sites, the basic goal seemed to be to achieve a pre-pandemic project state, in which the needs of the various women groups could be addressed in the best possible way (e. g., closed windows and doors, no participant limits, no additional costs for rapid tests). Also, it seemed unclear to some municipalities whether the course revenues would cover the course costs at times when there was a participant limit or the need for a 3G or 2G status.

“The problem is, now [autumn/winter 2021] only six women can take part in each course and we have to see whether the costs, because it is quite expensive, also the rent [for the room], whether it pays off now”. (Interview community B)

Even the option of free sessions to attract new participants could not always be guaranteed under COVID-19 regulations. However, such offers seem essential for participant recruitment in order to sustainably maintain the project.

“You can't just send people to the classes like we used to, where we'd say: ‘Well, the training will take place at this time. And if you like it, then you just join in’. Everything is limited in terms of space and the number of people”. (Interview community C)

“What effects it [pandemic] will have [on course participation] in the medium term, i. e. when more women are allowed to take part in the classes again, I don't think we can estimate that yet”. (Interview community B)

Due to too many forced changes and adjustments to the project, the program lost a significant part of its original character and thus made significant losses in reach and efficacy. Nevertheless, according to the coordinators, women who were able to continue their participation in BIG despite the adaptations made, it seemed possible to achieve similar positive effects through participation as it was reported before the pandemic. From the coordinator’s perspective, those women who were able to participate in the classes during the pandemic seemed to benefit in a similar way from the exercise sessions and the social exchange with other women. Participating women reported back to the coordinators, that the time spent at BIG was a break from their stressful daily lives and an opportunity to do something for themselves and reconnect with pre-pandemic daily structures, such as the regular participation on a PA class and to socialize with others.

“Psychologically seen, it was very important. For mental health. They [participating women] always had like-minded people to talk to. In the midst of social distancing, there was just a small island of normality. Which was very important for the women. Also, a piece of self-determination. Because every time they say, 'I'm going to BIG now,' they also take time and say, 'I'm out of here, out of the apartment, out of the home office, out of homeschooling. I'm going to do something for myself'". (Interview community A)

## Discussion

The present case study reports on project coordinator’s experiences with maintaining and adapting a low-threshold PA program for women in difficult life situations (BIG) during the COVID-19 pandemic and the factors influencing their resilience capacities. While there has been much research on the resilience of healthcare workers (e.g., physicians, nurses) during the COVID-19 pandemic [44], relatively little is known about resilience capacities of community project staff coordinating PA promotion programs for deprived population groups. The current study offers new insights into factors influencing resilience capacities in times of crisis to overcome those unfavorable circumstances and promote PA even in times of the COVID-19 pandemic.

### Resilience as key factor to booster crisis management and adaption to new situation

All six sites showed a high level of willingness to adapt to the pandemic situation and to maintain the BIG project as best as possible. Our results emphasize that resilience is not only an individual resource for project coordinators to cope with suddenly occurring stressful situations but also an important resource on a community level, for example through the unbureaucratic and flexible provision of financial and material resources, the facilitation of flexible working hours and new working methods, such as streaming PA-classes and use new communication channels to stay in contact with participants and trainers.

Overall local project coordinators acted as “champions” within their community organizations, as they acted proactive, were able to anticipate problems occurring due to new COVID-19 regulations and communicated in various ways with trainers and stakeholders about the best ways to solve these issues. There is evidence to suggest that such champions may be important facilitators to successfully implement and sustain (community) health promotion programs and strategies [45]. In most cases in our study, the successful handling of the acute stressors (e.g., closure of all classroom courses, contact follow-up, communication of new arrangements with women) which were caused by the constantly changing regulations seemed to lead to positive adaption and growth, which led to a strengthening of personal resilience of the coordinators. Further, those coordinators, whose work routine was characterized by intense relationship management with participants and trainers, demonstrated higher levels of adaptive and absorptive resilience capacities, than those with less social cohesion. These results suggest, that the positive feedback from the participating women about the value of the BIG community and the positive effects of (online) class participation during the pandemic, gave the coordinators a sense of the significance in their work. Findings from Lagios et al.’s [46] longitudinal studies confirm the importance of employees perceiving meaningfulness and impact of work, affective job commitment and satisfaction, especially in times of social isolation induced by COVID-19 (e.g., caused by home office). In our study the coordinators’ work was characterized by a high degree of self-organization, as coordinators mainly work alone without a team and are self-reliance for their areas of responsibility. Successfully handling this high workload and the complexity of the sudden crisis seemed to strengthen the coordinators’ self-efficacy and could thus lead to cope better with the challenges they faced at that time. Similar behavioral patterns were reported as crucial for adaptive capacity in a qualitative study regarding island communities and disaster resilience [47]. Studies suggested that coping mechanisms may be associated with emotional regulation, personality traits and a stable relationship with early caregivers [48, 49].

At a community level, important resources for strengthening adaptive, absorptive and anticipatory resilience capacities were job security of the coordinators, sufficient financial resources coordinators could use to adapt classes, as well as a supportive working environment to act flexibly according to current pandemic regulations. Those findings are in line with previous studies that demonstrate the great importance of community or organizational support to enable individuals to continually develop resources to adapt to acute stressors [50]. Such support can be the provision of financial resources, job security and trust [51]. In line with previous studies, supportive leadership, a good working climate, participation in decision-making or clear organizational communications seemed crucial to support health promotion projects to manage a sudden crisis [50]. As suggested from research from former crisis like the sudden spread of the Ebola virus, trust and trusted communication channels within a community, as well as strong social ties among community members who are working together to cope with a sudden crisis may be further important facilitating factors for community resilience [52].

### Resilience can only contribute to maintaining low-threshold PA programs to a limited extent

Our study also shows the limitations of resilience capacities. Even when individual and community resilience are established, not all communities were equally able to cope with the pandemic situation. Some sites were able to recover more quickly from unfavorable circumstances (e.g., lockdowns, hygiene regulations) than others. This was often due to more difficult circumstances, which they often could not influence themselves, such as lack of willingness to vaccinate among participants (community E), the missing provision of working materials and flexible working hours to stream classes online (community D) or the lack of available space to hold swimming lessons (community B). The consequence was that some women groups could no longer be addressed with BIG classes. A permanent transformation as envisaged in the WHO model, did not seem to be intended by the communities as the adapted classes lost a great deal of their low-threshold. And therefore, some women’s groups could no longer be reached. This can also be seen by the fact that the course programs for summer and fall 2022 contained almost no online or hybrid formats, but were overwhelmingly similar to pre-pandemic programs. However, the majority of these communities seems to be better prepared in case of future unfavorable events as they felt capable of adapting their PA and social activity programs again if needed to maintain the basic structures of the project.

Finally, the results of our study also indicate that maintaining a physical activity program for women in difficult life situations is highly relevant in times of crisis and can have a public health impact, during the pandemic. The coordinator indicated that the women who were able to participate in the adapted (i. e., online, outdoor) classes benefited from the participation. For example, (virtually) meeting with other women, doing something for themselves, taking “a time” out from the pandemic everyday life were seen as important aspects to promote the women’s mental health. Similar positive effects have also been reported by other studies. For example, a study by Luo et al. from 2022 showed that physical activity interventions could significantly increase the mental health of university students during the pandemic [53]. A structured park-based physical activity intervention in Italy seemed to foster the mood, self-control, well-being and vitality of the female participants [54]. Ramji and colleagues evaluated a Swedish community-based participatory research project, which similar to the BIG project, started with a physical activity intervention for women living in socially disadvantaged neighborhoods to strengthen the women’s empowerment before the pandemic [55]. Participating women reported that the physical activity project helped them to overcome frustration resulting from problems related to their past and current living situations and that they were convinced that staying physically active during the pandemic positively influenced their mental health. Further, participating in the intervention helped these women to feel included and as part of a larger group. The contact with other group members was also seen as important source of health-related information, which were rated as particularly important during the pandemic [55]. The results of this Swedish study, which overlap with our study results, emphasize once again that the chosen participatory project approach combined with physical activity classes could be an effective tool to reach women in difficult life situations and to generate positive health effects even in time of crisis, such as during the pandemic.

## Strengths and limitations

Our case study provides new insights into factors affecting community resilience to continue low-threshold PA promotion programs for women in difficult life situations during a sudden crisis. Given the ongoing global pandemic situation, the findings of this case study may help existing and future community-based participatory PA projects to respond to the demands of unpredictable events and to provide local structures and initiatives that act protectively on the sustainability of a project (e.g., sufficient funding for project staff, flexibility in project management and implementation).

In our study, we focus on the WHO’s concept of resilience capacities. As a result, we may have disregarded other dimensions that this model does not cover. Further, resilience is crisis and context-specific. Thus, it cannot be conclusively determined whether the resilience capacities presented also apply to other unfavorable events not associated with the COVID-19 pandemic, or whether other dimensions would be relevant beyond that. Results relate specifically to cultural and structural characteristics in Germany. Results may not be transferable to other countries and cultural backgrounds. Further, our study did not explicitly consider the perspectives of participants or trainers, which in terms of data triangulation, might have brought further informative insights to the research questions.

The triangulation of different data sources strengthened the analytical process of the study as the different sources complement each other well. The different timing of the informal phone calls and the interview, for example, provided a comprehensive and longitudinal picture of the different sites during the first 18 months of the COVID-19 pandemic.

## Practical implications

At a policy level, community institutions need to be aware that sustainable, effective and needs-based PA promotion should be given a higher priority in community policy, also in times of crisis. This requires reliable funding for project coordination, as well as the provision of flexible funds so that in times of crisis, expenses necessary for the transformation of a project can be made quickly and unbureaucratically. Further, our study implicates the need to provide additional resources for those offering exercise classes such as free rapid-tests or the provision of masks to strengthen the low-threshold of a program and to reach those with low income. Learning from experiences of clinics that have initiated resilience programs for health workers during the pandemic, can also support communities and their employees to cope with crisis. A scoping review from Rieckert et al. [56] for example emphasizes the value of a blame-free working environment with open communication channels, where employees can share information, experiences and good practice examples to strength natural coping mechanisms, such as acceptance or positive framing. Strengthening employees’ resilience seems worthwhile on an organizational level, as the current literature shows evidence on resilience positive effects on work engagement and further plays a mediating role between depression and burnout [44]. Therefore, counseling sessions or cognitive behavioral sessions seemed to be useful interventions to translate employees’ resources into workplace resilience [44].

## Conclusion

This case study provides new in-depth insights into a PA project for women in difficult life situations that was implemented before and maintained during the COVID-19 pandemic. The study provides a heterogenous and comprehensive picture of the associated challenges at the different sites and which facilitators and barriers affected the project coordinators’ resilience, especially their adaptive, absorptive, anticipatory and transformative resilience capacities. Findings highlight the great importance of permanent funding of project coordinators’ workplace as well as additional personnel and financial measures to absorb and adapt to the crisis. Also, a working environment based on trust and ownership, maintaining contact with the women, and the network of trainers and stakeholders were important factors in building resilience capacities. A sense of purpose in one's work was also important for coping with the additional stressors. The present results may not only be relevant in light of the COVID-19 pandemic. The findings provide suggestions on how to maintain health promotion projects permanently in a community through resilience. Strengthening of resilience should thereby always be considered at a policy level, as future national and global crises are to be expected in times of war, nuclear threats and the climate change.

## Data Availability

The dataset generated and analyzed during the current study are not publicly available due to data protection regulations of the study country in order to protect the privacy of the study participants. Deidentified data are available from the corresponding author on reasonable request.

## Abbriviations

BIG: Bewegung als Investition in Gesundheit (Movement as investment in health)
PA: Physical activity
SARS-CoV-2: Severe Acute Respiratory Syndrome Coronavirus 2
WHO: World Health Organization
